# Supplementary material for: The prosubiculum in the human hippocampus: A rostrocaudal, feature-driven, and systematic approach
Source: J Comp Neurol. Author manuscript; Available in PMC 2025 Mar 1. (PMC11060218; doi:10.1002/cne.25604)
Supplement: Supinfo [file NIHMS1973107-supplement-Supinfo.zip › Rosenblum_table_1.docx]

| **Case** | **Age** | **Sex** | **Brain Weight (g)** | **BB Stage** | **Hemi** | **PMI (hrs)** | **Cause of Death** |
| --- | --- | --- | --- | --- | --- | --- | --- |
| 1 | 45 | F | 1215 | 0 | LH | 24 | Ischemic Renal injury |
| 2 | 49 | M | 1300 | 0 | LH | 3 | Liver cirrhosis |
| 3 | 61 | M | 1310 | 0 | RH | 23 | Sepsis |
| 4 | 67 | M | 1380 | 0 | RH | 48 | Lung cancer |
| 5 | 68 | M | 1320 | 0 | RH | 17 | Malignant mesothioloma |
| 6 | 79 | M | 1200 | I | LH | <24 | Surgery Complication |
| 7 | 60 | M | 1166 | II | RH | <24 | Liver failure |
| 8 | 60 | F | 1328 | II | LH | 2 | Adenocarcinoma |
| 9 | 75 | M | 1187 | II | LH | 24 | Vascular disease |
| 10 | 84 | F | 1221 | II | LH | <24 | Heart failure |

**Table 1.** **Demographic information for cases used in this study.** Cases sorted by Braak and Braak stage and further sorted by age within respective stage. Abbreviations: BB Stage = Braak & Braak staging, F= Female, g=grams, hrs= hours, LH = left hemisphere, M= Male, PMI= post-mortem interval, RH = right hemisphere
