# Supplementary material for: The prosubiculum in the human hippocampus: A rostrocaudal, feature-driven, and systematic approach
Source: J Comp Neurol. Author manuscript; Available in PMC 2025 Mar 1. (PMC11060218; doi:10.1002/cne.25604)
Supplement: Supinfo [file NIHMS1973107-supplement-Supinfo.zip › Rosenblum_Manuscript_20240112_clean.docx]

**The prosubiculum in the human hippocampus: a rostrocaudal, feature-driven, and systematic approach.**

Emma W. Rosenblum ^1^, Emily M. Williams^1^, Samantha N. Champion^3^, Matthew P. Frosch^3^, Jean C. Augustinack ^1,2^

**Author affiliations:**

1 Athinoula A. Martinos Center, Massachusetts General Hospital, Department of Radiology, Charlestown, MA, USA

2 Harvard Medical School, Boston, MA, USA

3 C.S. Kubik Laboratory for Neuropathology, Massachusetts General Hospital and Harvard Medical School, Boston, MA, 02114

**Correspondence to:**

Jean C Augustinack

Full address: ​​Department of Radiology, Athinoula A. Martinos Center for Biomedical Imaging, Massachusetts General Hospital, Building 149 Thirteenth St. Room 2301, Charlestown, MA, 02129, USA

E-mail: jaugustinack@mgh.harvard.edu

**Running title: Prosubiculum in human hippocampus**

**Keywords:** cytoarchitecture, parcellation, hippocampal, pyramidal neurons, subfield, Nissl, CA1, Subiculum

**Data Availability**

Representative Nissl staining for every case presented in this study have been included in the figure material, but further inquiries should be directed to the corresponding author upon reasonable request.

**Ethics Statement**

The studies involving human participants were reviewed and approved by the Internal Review Board at the Massachusetts General Hospital. The patients/participants provided their written informed consent to participate in this study.

**Acknowledgements**

We are grateful to the brain donors and their families for their generous support, for without them this research would not be possible. We are thankful for Dr. Peter Davies and the tau antibody gift.

**Funding**

Grant support for this research was provided by the National Institute of Health: R01AG057672 and RF1AG072056.

**Conflicts of Interest**

The authors declare that the research was conducted in the absence of any commercial or financial relationships that could be construed as a potential conflict of interest.

**Key Points:**

- Small neurons, lightly stained neurons, clustered neurons and a cell sparse zone define key cellular features for prosubiculum.
- Small pyramidal neurons is the most prominent feature of the prosubiculum, while the other architectonic features appear heterogeneously.
- The prosubiculum is an independent subfield in the hippocampus based on its unique cytoarchitecture in the human brain.

**Abstract**

The hippocampal subfield prosubiculum (ProS), is a conserved neuroanatomic region in mouse, monkey and human. This area lies between CA1 and subiculum and particularly lacks consensus on its boundaries; reports have varied on the description of its features and location. In this report, we review, refine, and evaluate four cytoarchitectural features that differentiate ProS from its neighboring subfields: 1) small neurons 2) lightly stained neurons, 3) superficial clustered neurons, and 4) a cell sparse zone. ProS was delineated in all cases (n=10). ProS was examined for its cytoarchitectonic features and location rostrocaudally, from the anterior head through the body in the hippocampus. The most common feature was small pyramidal neurons, which were intermingled with larger pyramidal neurons in ProS. We quantitatively measured ProS pyramidal neurons, which showed (average, width at pyramidal base = 14.31 μm, n=400 per subfield). CA1 neurons averaged 15.57 μm and Sub neurons averaged 15.63 μm, both were significantly different than ProS (Kruskal-Wallis test, p < .0001). The other three features observed were lightly stained neurons, clustered neurons, and a cell sparse zone. Taken together, these findings suggest that ProS is an independent subfield, likely with distinct functional contributions to the broader interconnected hippocampal network. Our results suggest that ProS is a cytoarchitecturally varied subfield, both for features and among individuals. This diverse architecture in features and individuals for ProS could explain the long-standing complexity regarding the identification of this subfield.

**INTRODUCTION**

While the boundaries of the hippocampus as a whole are well recognized (Boccardi, Bocchetta, Apostolova, et al., 2015; Boccardi, Bocchetta, Ganzola, et al., 2015), the smaller subfields remain to be harmonized (Wisse et al., 2017; Yushkevich et al., 2015). One example is the boundary between the subiculum (Sub) and CA1, which has been especially variable among multiple reports. In a study comparing the hippocampal subfield parcellations by 20 different groups (same MRI), the boundary between CA1 and Sub showed the most disagreement (Yushkevich et al., 2015). The participating groups used a variety of factors or criteria including differences in MRI contrast, cellular organization patterns, geometric and anatomical markers or the boundaries described in Duvernoy’s book (Bender et al., 2013; Duncan et al., 2014; Kerchner et al., 2012; Kirwan et al., 2007; La Joie et al., 2010; Malykhin et al., 2010; Mueller et al., 2007; L. Wang et al., 2003; Winterburn et al., 2013; Wisse et al., 2012; Yushkevich et al., 2015; Zeineh et al., 2012). Two branchpoint categories emerged for CA1/Sub boundary: 1) some groups assigned the boundary more medially 2) and others designated it more laterally (Yushkevich et al., 2015). This variability at the CA1 and Sub boundary partially relates to the area known as prosubiculum (ProS).

The term prosubiculum was introduced by Oksar and Cécile Vogt (1919) but they did not define this subfield beyond describing it as a modified subiculum. Lorente de Nó, Ramon y Cajal’s student, wrote extensively about ProS and believed that ProS could be differentiated by the termination of the stratum radiatum (Lorente de Nó, 1934). Lorente de Nó reported that ProS was divided into three subparts, ‘a’, ‘b’, and ‘c’. Rosene and Van Hoesen (1987) described ProS in the monkey and human brain and noted its distinction, but only components ‘b’ and ‘c’ as described by Lorente de Nó were included in their ProS parcellation. Rosene and Van Hoesen were the first to specifically describe the cellular features of the ProS, noting its small neurons and superficial cell clustering in the pyramidal layer. Insausti and Amaral questioned whether ProS stands on its own as a subfield (Insausti and Amaral, 2004) and regarded the ProS as a transition zone between CA1 and Sub, based on a lack of functional differences. Adler et al. and Steve et al. both did not include ProS (Adler et al., 2014; Steve et al., 2017), while Ding and Van Hoesen (2015) parcellated ProS using NeuN and confirmed the finding of small cells and clustered cells within ProS that were first described by Rosene and Van Hoesen (1987). Palomero-Gallagher et al. (2020) elucidated the receptor architecture of ProS, highlighting it as a subfield. Williams et al. further described the parcellation of ProS and noted the neurons as small, lightly stained neurons that cluster superiorly (Williams et al., 2023).

The issue with ProS and its boundaries shows that neuroanatomical location matters, as noted in several neuroscience commentaries (Amunts et al., 2007; Devlin & Poldrack, 2007; Orban & Vanduffel, 2007; Passingham, 2007; Toga & Thompson, 2007; Turner & Geyer, 2014; Tzourio-Mazoyer et al., 2007; Van Essen & Dierker, 2007). The goal of this study was to review, refine and critically evaluate the explicit cytoarchitectural features of ProS. We identified four prosubiculum features – small pyramidal neurons that intermingle among larger neurons, lightly stained neurons, clustered neurons, and a cell sparse zone. The first three features have been loosely described by previous neuroanatomy studies and a cell sparse zone is a novel feature observed in this report. By evaluating and comparing these features across our ten hippocampal samples rigorously, we conclude that ProS can be identified across samples, although with a degree of inter-individual variability.

**METHODS**

**Tissue Samples**

Ten human hippocampi samples were evaluated in this study. **Table 1** lists the demographics of all cases. The brain hemispheres were obtained by the Massachusetts General Hospital Autopsy Service and were fixed in 10% formalin for a minimum of two months. The brains were collected according to the guidelines of Massachusetts General Hospital’s Internal Review Board. The postmortem intervals were less than 24 hours for all cases except for one (case 4), which had a 48 hour postmortem interval. The ages ranged between 45-84 years old and included seven males and three females. Total brain weights ranged from 1187 and 1380 grams (mean±stdev = 1262.7 grams ± 73.0) and included four right and six left hemispheres. The cases were all cognitive controls with no history of memory impairment. Brain sections were immunostained for tau pathology using CP13 (generous gift by Dr. Peter Davies) and staged according to Braak and Braak staging (Braak et al., 2006; Braak & Braak, 1991). The phosphorylated tau pathologic diagnoses according to Braak and Braak staging of the cases were: five controls, one Braak and Braak stage I and four Braak and Braak stage II. Vascular strokes and/or microinfarcts were not observed. Only one case, the oldest case in our sample set, case 10, was positive for CP13-reactive neuritic plaques; the remaining cases showed no amyloid.

**Blocking, Sectioning Procedures**

Samples were stored in periodate-lysine-paraformaldehyde at 4° C before blocking the medial temporal lobe. All brain samples were blocked in the coronal plane. Blocked medial temporal lobe samples were cryoprotected (20% glycerol, 2% dimethyl sulfoxide) for a minimum of one month before sectioning. The samples were sectioned serially in the coronal plane at 50 μm with a sliding freezing microtome (Leica SM2000R, Leica Biosystems Inc Buffalo Grove, IL USA) with a customized stage. All sections were collected, stored in cryoprotectant solution and frozen at -20°C prior to staining experiments.

**Histology Procedure**

Sections were rinsed to remove cryoprotectant and mounted on gel coated glass slides, dried overnight, and stained with thionin for Nissl substance. The staining procedure involved the following steps: defatting (chloroform: 100% ethanol, 1:1), an immersion in 50% ethanol, pretreatment (acetone: acetic acid: 100% ethanol: double distilled water, 1:1:1:1), staining (5% thionin, 1.36% sodium acetate stock and 0.6% acetic acid stock) and differentiation (70% ethanol with 5-10 drops of glacial acetic acid). The slides were dehydrated in increasing ethanol concentrations, cleared with xylene and coverslipped using Permount (Fisher Scientific, NH USA). Silver staining for myelin was carried out according to the Gallyas method (Gallyas, 1979).

**Rostrocaudal Levels of the Hippocampus**

We assessed ProS criteria at nine anterior-to-posterior hippocampal levels in this study including: 1) *genu*, 2) *genu-pes,* 3) *pes,* 4) *pes-dentate gyrus,* 5) *full dentate gyrus,* 6) *separated dentate gyrus,* 7) *X-region,* 8) *uncus-body* and 9) *body* (Williams et al., 2023). Briefly, the *genu* was defined as the most anterior and smallest level of the hippocampus before any digitations (pes) emerge. The *genu-pes* is the level where at least one pes appears, and the shape of the hippocampus is a combination of *genu* and *pes*. This level is followed by the *pes* level, which has multiple pes, and the hippocampal sulcus has fully opened. The *pes-DG* level begins when small slivers of dentate gyrus (DG) appear. *Full DG* represents where the DG is at its largest medially to laterally and CA4 is within the DG. At the next level – the *separated DG* – the DG separates into two structures but tissue is still physically connected. The *x-region* occurs where the lateral part of the hippocampal head and the medial uncus begins to form two discontinuous structures, but the pyramidal layer is still contiguous forming an X. The *uncus-body* level is immediately before the head/uncus ends and/or the uncus is fully separated from the hippocampal body. The last level is the hippocampal *body*. The hippocampal tail was not included in this study.

**Tissue selection and analysis**

Eighteen histologic sections per case were sampled and analyzed for ProS features (180 slides total). Histology sections were at least 500 µm apart and at one of nine anterior-posterior hippocampal levels. Two histology sections were selected at each anterior-posterior hippocampal level, except the x-region (one slide, due to limited size of this region) and the body (three slides, given the large span of this level). Stained sections were assessed using an Olympus BH-2 double headed microscope (Precise Instrument, Hansen MA) and a Keyence (Osaka, Japan) digital microscope (BZX800). The selected sections were evaluated using the Olympus BH-2 double headed microscope to identify the overall cytoarchitecture and the subiculum, ProS and CA1 region. We evaluated the microscopic features along the cortical ribbon spanning specifically from CA2 to presubiculum (to fully encompass regions CA1, ProS and Sub). The boundaries were recorded, and the slides were digitized with a Keyence digital microscope (Osaka, Japan) at 4x magnification where the parcellated boundaries were digitally saved (GIMP, visualization software in Unix).

**Hippocampal Subfield Parcellation**

The hippocampal subfield parcellation was based on previous studies (Lorente de Nó, 1934; Rosene and Van Hoesen, 1987, Ding and Van Hoesen, 2015, Insausti and Amaral, 2004, Williams et al. 2023) but the prosubiculum, and its neighbors, subiculum and CA1 were the focus here.

**Prosubiculum Parcellation Protocol**

We constructed a protocol to identify ProS based on a review of the existing literature (Table 2) and novel observations. We settled on four features 1) small, narrow pyramidal neurons, 2) pyramidal neurons that display lighter staining or display less chromophilia than its immediate neighbors, 3) neurons that cluster together in a dense strip superficially in the pyramidal layer (Rosene and Van Hoesen, 1987, Ding and Van Hoesen, 2015), and 4) a sparse zone within the middle of pyramidal layer (centrally within pyramidal layer, not superficial and not deep). The presence of features 1 and 2 were determined relative to the neighboring subfields on the same tissue section (same slide and same case). In other words, for neurons to be considered small, they must show a narrower soma than those of CA1 and Sub in that particular histologic section. Chromophilia means staining loving and produces rich, darkly stained neurons; thus, the lack of chromophilia results in lightly stained neurons. The clustered neurons appear either continuous across the entire length of the subfield or may be intermittent clusters. The presence of features 3 and 4, clumped cells and a cell sparse zone respectively, were not present in CA1 and nor Sub. All four features apply to the neurons within the pyramidal layer of ProS. Neurons may be classified by one feature or multiple (i.e. small neurons, or lightly stained neurons or both features). Our approach was similar to most parcellation and pathology studies weighing multiple features each on a binary basis (present or absent), thus, in a sense, a simplistic semi-quantitative approach. The presence or absence of each feature was noted while parcellating.

**Neuron size, quantitative measures**

The pyramidal neuron size was manually measured in CA1, ProS, and Sub regions. Neuronal widths were measured at the base of the pyramidal neuron, immediately superior to the basal dendrites. The quantitative measures were collected in CA1, ProS, and Sub at four anterior-posterior hippocampal levels: pes, full DG, x region and body (Williams et al. 2023). Using the Stereoinvestigator software attached to a Nikon 80i microscope, quantitative measures were collected at 200x magnification (20x objective). The software was utilized to determine 10-15 systematically random counting frames (100 µm x 100 µm) with two inclusion and two exclusion lines. Sampled neurons must have exhibited 1) a discernible nucleus, 2) did not overlap with other neurons, and 3) the nucleus did not intersect with the exclusion lines of the counting frames. Ten neurons per subfield were measured, resulting in n=400 per subfield with three subfields (n=1200 total neurons).

**Statistical Analysis**

Given the descriptive nature of the evaluation of features, the absence or presence of each feature was tallied for each sample and no formal inferential comparisons were performed. On the quantitative neuron size measures, Shapiro-Wilk tests were performed to determine the normality of each group. A Kruskal-Wallis one-way analysis of variance (ANOVA) was significant and post hoc analysis with Dunn’s multiple comparisons tests was used. The level of significance was set at p < 0.05. Statistical analysis was performed in Prism v9.1 (Graphpad).

**RESULTS**

The microscopic view illustrates the small, narrow pyramidal neurons in ProS in comparison to those in CA1 and Sub (**Figure 1 - A to O)**. **Figure 1A** shows neuron outlines that correspond to **Figure 1B.** **Figure 2** displays the quantitative measures of neurons in Sub, ProS and CA1**.** ProS neurons averaged 14.31 μm, CA1 neurons averaged 15.57 μm, and Sub neurons averaged 15.63 μm (Figure 2), which demonstrates the significant differences among the groups (CA1, ProS and Sub neurons (Kruskal-Wallis test, p < .0001). Further testing through a Dunn’s multiple comparisons tests showed significant differences between ProS and CA1 or Sub, respectively (both p <.0001). **Figure 3 (A to J)** shows the mesoscopic view of ProS and allows the visualization of contrast between neighboring subfields on the same section but also shows clustered neurons, the sparse cell zone and lightly stained neurons. In the following sections, each feature will be described. **Figure 4** **(A to J)** depicts a macroscopic view of the ten cases at the level of the anterior body, which enables visualization of ProS boundaries with its neighbors. **Figures 3 and 4** are arranged in the same case order as in **Table** **1**. ProS was located as the hippocampus curves dorsally toward the temporal horn of the lateral ventricle and ProS was always observed between subfields CA1 and Sub (**Figure** **4**). While the relative location of the ProS remained consistent between CA1 and Sub, the width of the cortical ribbon of the ProS varied from case to case. Some cases have a relatively narrower ProS medially-to-laterally along the cortical ribbon (**Figure** **4B, D, G, J**) compared to cases that showed wider territory (**Figure 4A, E, I**).

All four cellular features (small pyramidal neurons, lightly stained neurons, superficially clustered neurons, sparse cell zone) were observed across the 10 cases. The prevalence and combinations of these features, however, varied substantially.

Small Neurons

**Figure 1** shows the first criteria, small neurons, that appear distinctly smaller and narrower than neurons outside of the ProS boundaries. The observation of small neurons was a consistent feature for ProS. Yet, neuron size varied within each ProS subfield, at times revealing a gradient with smaller neurons superficially and larger neurons deeper in the pyramidal layer. We observed that the small neurons appeared intermingled with larger neurons within the ProS subfield. Thus, ProS did not only show small cells, but was differentiated from the surrounding subfields by small pyramidal neurons that were intermingled with some larger pyramidal neurons.

Lightly Stained Neurons (Lack of Chromophilia)

The second criteria, lightly stained neurons (the lack of chromophilia), appear in **Figure 1B, E, K** and often concurs with the neuron size**.** Similarly, as with the “small neurons” feature, the ProS region showed a range of varying staining intensity. Lightly stained neurons were best observed at the microscopic view and to some degree the mesoscopic view but it requires comparison between ProS and its neighboring subfields (**Figures 1 and 3**).

Clustered Neurons

Clustered neurons appeared as a superficial, compact strip or clump of dense neurons that were typically more chromophilic than underlying neurons (**Figure 3B, E, H, I**). The presentation of the clustering was heterogeneous, for both neurons and layers. In some instances, we observed clustered neurons were present across the entire medial to lateral span of the subfield (**Figure 3B**) or just part of the subfield (**Figure 3E, H, I**). The packing density (or pattern) of the clustered neurons often differed among cases, demonstrated in **Figures 3B and E**; note even though the densities between the two instances of clustered neurons differ, still both examples were considered clustered. These examples illustrate more densely clustered neurons than the surrounding neurons.

Sparse Cell Zone

We observed two presentations of the sparse cell zone: either no cells (**Figure 3H**) or an area with much lower packing density (**Figure 3E, I)**. The cell sparse zone was at times observed beneath clustered neurons (**Figure 3E, H, I**). The cell sparse layer, if present, was in the middle one third depth of the pyramidal layer within the subfield (**Figure 3E, H, I**). The sparse cell zone was observed in control cases and Braak and Braak I and IIs.

Collective Appearance of Prosubicular Features

**Figure 5** serves as a companion figure to **Figure 6**. **Figure 5** shows a gross specimen of the human hippocampus with anatomical landmarks (**Figure 5A**), coronal lines of cut (**Figure 5B**) and coronal section schemas at four anterior-posterior levels (**Figure 5C**). The coronal lines of cut in Figure 5B show the anatomical locations sampled in **Figure 6**. We compiled the location and frequency of features observed throughout the ProS in all cases in our sample set in **Figure 6 (A to J)**, which consists of a matrix for every case. The case order is organized in the same sequence as **Table 1**. Each matrix shows the presence or absence of the four features on each analyzed section. Due to the binary nature of our parcellation protocol – present or absent – these matrices show a conservative representation of features. For example, **Figure 3C** has slightly more clustered neurons than other non-clustered-neurons cases; however, to meet the clustered criteria, neurons must be located superiorly within the pyramidal layer and appear somewhat continuous. The beginning of ProS is indicated by at least one ProS feature being present. The matrices show the ProS typically begins at the level of the *genu-pes* and continues consistently through the *body* of the hippocampus. Three cases, cases 2, 5 and 8, ProS began at the level of the *genu* **(Figure 6B, E, H),** specifically case 5, in which ProS was present in the most anterior genu section analyzed. The most common feature was small neurons (blue), which was found in all cases and present in 93% of the slides with ProS present (excluding the anterior slides before the beginning of ProS). The next two most common features were lightly stained neurons (pink) and clustered neurons (orange), which appeared at a similar frequency in Nissl sections with ProS present (39% and 38%, respectively). Related, both features appear intermittently. For example, the clustered cell feature appeared in every case but was present on one section (case 1, **Figure 6A)** or up to sixteen Nissl sections (case 5, **Figure 6E**). The least common feature was the cell sparse zone (green), which appeared in only 22% of Nissl stained sections with ProS. Often, the cell sparse layer was present on sections that also show the clustered feature. These two features run together.

Phosphorylated Tau Immunostaining

To assess whether the cell sparse zone was due to tau pathology, we evaluated ProS with phosphorylated tau immunostaining. The control and Braak and Braak I (BBI) cases all showed isolated tangles (1-3 per section). The Braak and Braak II (BB II) cases contained approximately a dozen per section in ProS, and the two oldest BBIIs had substantially higher tangles per slide, approximately 20 tangles per slide in ProS. Notably, the location of the tangles within the ProS pyramidal layer was noted and the phosphorylated tau tangles were distributed throughout the subfield (inferior to superior), not just where the sparse cell zone is located. A photomacrograph of a tau immunostained section from Case 9 (BBII) is shown in **Figure 7A**. **Figure 7 B-D** show tau tangles in Sub, ProS, and CA1, respectively. Note the small tangles intermingled with larger tangles in **Figure 7C**.

**Discussion**

The basic goal in this paper was to standardize the criteria to evaluate ProS and demonstrate its presence in the human brain. This report defines the four criteria that characterize the ProS, but also advances the parcellation protocol that evaluate cellular traits, as opposed to a lamina approach. In this study, we provide evidence for the existence the ProS subfield in the human hippocampus by tracking and demonstrating its cellular features: small neurons, lightly stained neurons, neuronal clumping, and a cell sparse zone. The small neurons feature dominated in all cases (**Figure 6**). The other three features appeared less consistently (**Figure 6**) and the matrices show their heterogeneity. ProS appeared throughout our hippocampal samplings from hippocampal anterior head to body, similar to previous reports (Barbas & Blatt, 1995; Ding & Van Hoesen, 2015, Wang & Barbas, 2018). The prevalence of these features did not follow any specific pattern. This demonstrated heterogeneity may be the reason for the past controversy in identifying the ProS.

ProS is a conserved neuroanatomic region among mouse, monkey and human (Ding, 2013). In both rat and monkey, similar features such as small and clustered neurons were used to distinguish ProS from its neighbors (Ding, 2013; Rosene & Van Hoesen, 1987). Like humans, the ProS in the monkey showed higher acetylcholinesterase staining than CA1 and Sub, while only acetylcholinesterase distinguished ProS from CA1 (but not Sub) in the rat (Rosene & Van Hoesen, 1987). Our report shows the comparative aspects of inter-individuality in the human brain.

Presubiculum (PreS) is the subfield that borders Sub medially. It is recognized by how its layer II clusters together (i.e. presubicular clouds), the presence of lamina principalis externa medially and small size of layer II neurons (Ding & Van Hoesen, 2015; Insausti & Amaral, 2004; Williams et al., 2023). Presubiculum shares an oblique boundary with subiculum as the PreS clouds overlay superiorly to the large underlying subicular pyramidal neurons. ProS parallels some similarities to PreS, such as small neurons superficially and ProS occasionally exhibits an oblique boundary with its neighboring subfields. The ProS exhibits small and narrow pyramidal neurons intermingled among more typically sized pyramidal neurons of subiculum and CA1 (Figure 1). The PreS neurons display some of the smallest neurons in the hippocampal formation. Presubicular pyramidal neurons encode for an animal's head direction, but it is not clear whether location was precise for presubiculum (Tukker et al., 2015) or nearby in the parahippocampal gyrus (Taube et al., 1990).

ProS and CA2 have a couple things in common. First, both regions, ProS and CA2, have been previously grouped together with larger, and possibly better understood neighboring hippocampal subfields, such as CA1 and CA3, respectively. As a result, ProS and CA2 have been referred to as transition areas. CA2 is a small subfield located between CA1 and CA3, which also has perforant pathway connectivity (Ding et al., 2010).

CA2 is known for its large, darkly stained pyramidal neurons, which sometimes clump inferiorly (Ding & Van Hoesen, 2015; Duvernoy, 2005; Insausti et al., 2023; Insausti & Amaral, 2004; Williams et al., 2023). This feature, neuronal clumping, is the second commonality that ProS and CA2 both exhibit, albeit superiorly (ProS) and inferiorly (CA2). Findings from animal models have suggested that the function of CA2 pertains to social memory (Hitti & Siegelbaum, 2014; Lehr et al., 2021; Oliva, 2022; Oliva et al., 2023). The function of prosubiculum in humans is unknown. Identifying and mapping ProS in the human brain in anatomical studies such as this one may help elucidate its function**.**

In regards to human studies that discuss ProS, our data agrees with Lorente de Nó (1934), Rosene and Van Hoesen (1987), Duvernoy (2005), Ding and Van Hoesen (2015), Palomero-Gallagher et al. (2020), and Williams et al. (2023) who included ProS in their parcellations. To identify ProS, Lorente de Nó (1934) relied on the disappearance of the stratum radiatum, Rosene and Van Hoesen (1987) and Williams et al. (2023) relied on cell size and organization, while Ding and Van Hoesen (2015) also cited differences in staining (NeuN) as well as similar cellular features to Rosene and Van Hoesen (1987). Duvernoy (2005) included ProS in his schemata depicting the hippocampal subfields boundaries, and even referenced the ProS controversy, but lacked a description of the ProS features or the criteria used to parcellate ProS. Conversely, Insausti and Amaral (2004) and Adler et al. (2014) did not include ProS in their parcellations. Insausti and Amaral (2004) wrote that ProS should be considered only a transition zone due to its lack of known functional differences. Adler et al. (2014) noted that the boundary between CA1 and Sub is “complex”, butalso noted it as limitation of their study. This discrepancy among publications shows the need for clear-cut features to accurately identify this region and our findings provides this.

The most common feature we found in the ProS was small neurons **(Figure 6)**. ProS neurons were significantly narrower than CA1 and Sub neurons when assessed qualitatively and quantitatively (**Figures 1 and 2**). This finding is in line with previous qualitative observations (Ding, 2013; Rosene & Van Hoesen, 1987). Small neurons represent a key part of ProS because we observed them in almost every Nissl section with ProS (**Figure 6**). Notably, we expand on this description of the small cells by noting an intermingling and varying of pyramidal neuron sizes within ProS. The small, intermingled neurons were even noted when manifested in tau tangles (**Figure 7C**). While the small neurons were differentiated in CP13 tau staining, this stain was not used for parcellating or viewing cytoarchitecture.

Lightly stained neurons lack chromophilia in Nissl staining compared to cells in the neighboring subfields **(Figure 1 B, E, H).** Lightly stained neurons were found at the same rate as the clustered neuron feature. Most likely, this feature stems from small cells, which contain less Nissl substance than larger neurons. It is important to note that these staining differences were not due to differences in staining quality, as neurons should only be compared to neurons within the same histological section.

Clustered neurons are defined by a higher packing density of larger, darkly stained neurons (**Figure 3B, E, H, I**)**.** This feature is distinct at the macro- and mesoscopic magnification. Past reports did not define this term, making it challenging to determine what the clumped cells looked like and what qualifies as “clustered.” The superior clustering was both denser than the other ProS neurons and those in the neighboring subfields. Wang and Barbas (2018) showed neuronal clustering superficially in the monkey prosubiculum using Nissl staining in their Figure 1C-D. The neuronal clustering in the monkey resembles the same clustering in the human brain demonstrated in this report. The monkey prosubiculum appeared throughout the rostrocaudal levels of the hippocampus, again similar to our findings in the human brain.

The least prevalent was the cell sparse layer or zone (**Figure 6**), which runs through the middle of the pyramidal layer when present (**Figure 3E and 3H**). The cell sparse zone is often found beneath the clustered cells. This feature was not previously described and is a novel finding in this work. We speculate that the cell sparse feature does not appear to be a result of tau pathology for two reasons. First, the cell sparse zone appears in cases regardless of Braak and Braak tau staging (in controls), and second, the tau pathology that manifests is not restricted to the middle sublayer of the subfield**.** Thus, the tau pathology was observed throughout the subfield with no pattern that relates to the cell sparse zone in Braak and Braak II cases. It could be hypothesized that the cell sparse zone is due to subclinical neuronal death unrelated to tau pathology, but this could be a topic for further research.

The CA1 and Sub features help determine the parcellation boundary lines too. Sub is known for larger, darker neurons, while CA1 is distinguished by large, sparsely distributed neurons that form a jagged inner border (Ding & Van Hoesen, 2015; Insausti & Amaral, 2004; Williams et al., 2023). Note, the arrowheads in **Figure 4B** point to examples of the jagged inner border of CA1. The subiculum routinely shows an oblique, overlapped boundary that occurs on both sides, its medial and lateral boundaries. This overlapping border is not unique to ProS and persists throughout the hippocampus and cortex. Often, an oblique boundary line between ProS and Sub captures this transition most accurately. Of course, most cortical boundaries do not create strict, easy lines between them. To clarify and further understand hippocampal subfield function in the future, qualitative boundary lines have been a necessary first step (Ding et al., 2016; Insausti et al., 1995, 2017; Olsen et al., 2019; Williams et al., 2023; Yushkevich et al., 2015)

Our findings demonstrate that the variability of features in ProS reflect a cytoarchitectonic heterogeneity. The size and location variation reported in this study is likely the result of the size and cerebral variability in the human brain. Though the location may appear to have substantial variation in the 2D histology sections, the variation stems from the human brain and its 3D cortical ribbon that is continuously shifting. **Figure 6** depicts the feature heterogeneity by summarizing the occurrence of each feature by subject and anatomical level. We did not observe differences based on age or postmortem interval in the current sample set. Given the sample size, formal comparisons of the distributions of these features were not performed. However, even without formal comparisons, there is noticeable variability in features among cases with similar demographics, such as the five control cases (cases 1-5, tau negative).

Other studies have determined additional defining characteristics of ProS through immunostaining and receptor-architecture studies. Rosene and Van Hoesen (1987) show high acetylcholinesterase staining in ProS when compared to CA1 and Sub, which was corroborated by Ding and Van Hoesen (2013)**,** Barbas & Blatt (1995) and Wang & Barbas( 2018). Though Bakst & Amaral (1984) did not explicitly parcellate prosubiculum, their figures show acetylcholinesterase staining between CA1 and Subiculum. Ding and Van Hoesen (2013) also reported tyrosine hydroxylase, neurotensin, SMI-32 and PV (parvalbumin) as markers to differentiate ProS when compared to Sub and CA1. Their work also showed differing gene expression of nnat (neuronatin) and htr2a to differentiate ProS from Sub and syntaxin, slc30a3, and neurod6 among others to differentiate ProS from CA1 (Ding and Van Hoesen, 2013). Ding 2015 furthered this analysis and showed ProS parcellated throughout the hippocampal head in Nissl, NeuN, PV. Palomero-Gallagher et al. (2020) identified receptor differences in ProS when compared to CA1 and Sub. CA1 showed higher AMPA, NMDA, GABA_A_, M2, α2, and 5-HT1A receptor densities and GABAA/BZ binding sites than ProS, while Sub showed to lower AMPA, NMDA, GABAB, 5-HT1A, and 5-HT2 than ProS. Palomero-Gallagher et al. also reported differing receptors densities between the molecular layer of Sub and ProS (2020). Taken together, these differences may suggest functional differences that support ProS’s delineation as an independent subfield. Here, we show architectonic markers were sufficient to parcellate ProS, but we also note that applying multiple markers, such as Nissl or NeuN, as well as specific markers (e.g. AChE) may provide the most accurate borders.

Saunders et al. showed distinct connectivity between ProS and amygdala, which could suggest a functional difference. Specifically, amygdala efferents from accessory basal nucleus (magnocellular only) and cortical nucleus project lightly to the superficial part of the pyramidal layer in the prosubiculum, which may suggest a functional role for the neuronal clusters observed cytoarchitecturally (Saunders et al., 1988). Rostral ProS was also shown to connect to medial, lateral and orbital prefrontal areas in the macaque monkey (Barbas & Blatt, 1995). A study in the mouse showed that prosubiculum connects with areas related to emotion, rewards, addiction, fear, and motivation (Ding et al., 2020).

This study has the potential to impact three fields. First, we added a criteria-based and multi-faceted evaluation of the human ProS, which will help standardize neuroanatomy. This report developed a more rigorous and architectonic feature-driven approach to evaluating brain areas. Second, these parcellations will help inform neuroimaging since MRI resolution and contrast does not allow visualization of the cellular architecture needed to parcellate the hippocampal subfields. The neuroimaging community relies on histology as the ground truth data to advise their parcellations (Adler et al., 2014; de Flores et al., 2020; Olsen et al., 2019). Validated parcellation can lead the way to discovering functional differences among subfields. Third, improving anatomical accuracy will impact potential consequences in neuroinformatics. Recently, deep learning approaches have been applied to histologic analyses (Niazi et al., 2019; Perosa et al., 2022, 2022; Waisman et al., 2021; Oltmer et al., 2023). Deep learning methods for segmenting pyramidal neurons may be applied to a high throughput experimental paradigm in the future, which may detect subtle neuronal loss and ultimately benefit disease treatment.

This study has some limitations. First, the sample set (n=10) is small; however, the amount of tissue territory covered, or the evaluation of hippocampal tissue in this study, was extensive. Second and related, this study has a limited number of tau-negative control cases (n=5). Unfortunately, this is the nature of postmortem studies. This could potentially mean some cases have cytoarchitectural changes due to aging. To limit cytoarchitectural changes due to pathology, we restricted samples to only cognitive control cases (no memory impairment) and preclinical stages.

This current work may set the foundation for three concepts: 1) the ProS existence, 2) the ProS location and its boundaries, and 3) ProS’s multiple and variable features. This work covers substantial territory to map the ProS, documenting its features. While other stains have been used to parcellate ProS, we used Nissl in our study to enable mass parcellation. Moreover, Nissl is inexpensive, widely available and can be used to quickly stain numerous serial sections, while visualizing the necessary cellular architecture (Amunts et al., 2013; Gómez-Isla et al., 1996, 1997; Insausti et al., 1995; Swanson, 2003; West et al., 2000). Nissl reveals the neuronal soma, which resembles NeuN (Gittins & Harrison, 2004). Staining for Nissl substance also allows us to evaluate the four identified features: small cells, lightly stained neurons, clustered neurons and the cell sparse zone. Immunostaining for NeuN does not show staining intensity (i.e. chromophilia from a stain) but instead generates a present or absent (binary) output. The value of this paper is in the regionality mapping, feature composition and inter-human variability of ProS in the human brain that these data have demonstrated. Parcellation depends on using a microscopic approach and multi-faceted features (not just one feature), weighing each one and then collectively weighing the four features (in this instance) together. This creates a criteria-based qualitative approach that helps to standardize the field of neuroanatomy. This standardization of the input data will benefit not only cortical neuroanatomy, but also deep learning methodology**.** This delineation clarifies boundary accuracy and may contribute to the discovery of functionality and other disease differences in the future.

References:

Adler, D. H., Pluta, J., Kadivar, S., Craige, C., Gee, J. C., Avants, B. B., & Yushkevich, P. A. (2014). Histology-derived volumetric annotation of the human hippocampal subfields in postmortem MRI. *NeuroImage*, *84*, 505–523. https://doi.org/10.1016/j.neuroimage.2013.08.067

Amunts, K., Lepage, C., Borgeat, L., Mohlberg, H., Dickscheid, T., Rousseau, M.-É., Bludau, S., Bazin, P.-L., Lewis, L. B., Oros-Peusquens, A.-M., Shah, N. J., Lippert, T., Zilles, K., & Evans, A. C. (2013). BigBrain: An ultrahigh-resolution 3D human brain model. *Science (New York, N.Y.)*, *340*(6139), 1472–1475. https://doi.org/10.1126/science.1235381

Amunts, K., Schleicher, A., & Zilles, K. (2007). Cytoarchitecture of the cerebral cortex—More than localization. *NeuroImage*, *37*(4), 1061–1065; discussion 1066-1068. https://doi.org/10.1016/j.neuroimage.2007.02.037

Bakst, I., & Amaral, D. G. (1984). The distribution of acetylcholinesterase in the hippocampal formation of the monkey. *The Journal of Comparative Neurology*, *225*(3), 344–371. https://doi.org/10.1002/cne.902250304

Barbas, H., & Blatt, G. J. (1995). Topographically specific hippocampal projections target functionally distinct prefrontal areas in the rhesus monkey. *Hippocampus*, *5*(6), 511–533. https://doi.org/10.1002/hipo.450050604

Bender, A. R., Daugherty, A. M., & Raz, N. (2013). Vascular risk moderates associations between hippocampal subfield volumes and memory. *Journal of Cognitive Neuroscience*, *25*(11), 1851–1862. https://doi.org/10.1162/jocn_a_00435

Boccardi, M., Bocchetta, M., Apostolova, L. G., Barnes, J., Bartzokis, G., Corbetta, G., DeCarli, C., deToledo-Morrell, L., Firbank, M., Ganzola, R., Gerritsen, L., Henneman, W., Killiany, R. J., Malykhin, N., Pasqualetti, P., Pruessner, J. C., Redolfi, A., Robitaille, N., Soininen, H., … Frisoni, G. B. (2015). Delphi definition of the EADC-ADNI Harmonized Protocol for hippocampal segmentation on magnetic resonance. *Alzheimer’s & Dementia : The Journal of the Alzheimer’s Association*, *11*(2), Article 2. https://doi.org/10.1016/j.jalz.2014.02.009

Boccardi, M., Bocchetta, M., Ganzola, R., Robitaille, N., Redolfi, A., Duchesne, S., Jack, C. R., Frisoni, G. B., & EADC-ADNI Working Group on The Harmonized Protocol for Manual Hippocampal Segmentation and for the Alzheimer’s Disease Neuroimaging Initiative. (2015). Operationalizing protocol differences for EADC-ADNI manual hippocampal segmentation. *Alzheimer’s & Dementia: The Journal of the Alzheimer’s Association*, *11*(2), Article 2. https://doi.org/10.1016/j.jalz.2013.03.001

Braak, H., Alafuzoff, I., Arzberger, T., Kretzschmar, H., & Del Tredici, K. (2006). Staging of Alzheimer disease-associated neurofibrillary pathology using paraffin sections and immunocytochemistry. *Acta Neuropathologica*, *112*(4), Article 4. https://doi.org/10.1007/s00401-006-0127-z

Braak, H., & Braak, E. (1991). Neuropathological stageing of Alzheimer-related changes. *Acta Neuropathologica*, *82*(4), Article 4. https://doi.org/10.1007/BF00308809

de Flores, R., Berron, D., Ding, S.-L., Ittyerah, R., Pluta, J. B., Xie, L., Adler, D. H., Robinson, J. L., Schuck, T., Trojanowski, J. Q., Grossman, M., Liu, W., Pickup, S., Das, S. R., Wolk, D. A., Yushkevich, P. A., & Wisse, L. E. M. (2020). Characterization of hippocampal subfields using ex vivo MRI and histology data: Lessons for in vivo segmentation. *Hippocampus*, *30*(6), Article 6. https://doi.org/10.1002/hipo.23172

Devlin, J. T., & Poldrack, R. A. (2007). In praise of tedious anatomy. *NeuroImage*, *37*(4), 1033–1041; discussion 1050-1058. https://doi.org/10.1016/j.neuroimage.2006.09.055

Ding, S.-L. (2013). Comparative anatomy of the prosubiculum, subiculum, presubiculum, postsubiculum, and parasubiculum in human, monkey, and rodent. *The Journal of Comparative Neurology*, *521*(18), Article 18. https://doi.org/10.1002/cne.23416

Ding, S.-L., Haber, S. N., & Van Hoesen, G. W. (2010). Stratum radiatum in CA2 of the hippocampus is a unique target of the perforant path in human and nonhuman primates. *Neuroreport*, *21*(4), Article 4. https://doi.org/10.1097/WNR.0b013e328333d690

Ding, S.-L., Royall, J. J., Sunkin, S. M., Ng, L., Facer, B. A. C., Lesnar, P., Guillozet-Bongaarts, A., McMurray, B., Szafer, A., Dolbeare, T. A., Stevens, A., Tirrell, L., Benner, T., Caldejon, S., Dalley, R. A., Dee, N., Lau, C., Nyhus, J., Reding, M., … Lein, E. S. (2016). Comprehensive cellular-resolution atlas of the adult human brain. *The Journal of Comparative Neurology*, *524*(16), 3127–3481. https://doi.org/10.1002/cne.24080

Ding, S.-L., & Van Hoesen, G. W. (2015). Organization and Detailed Parcellation of Human Hippocampal Head and Body Regions Based on a Combined Analysis of Cyto- and Chemoarchitecture. *The Journal of Comparative Neurology*, *523*(15), 2233–2253. https://doi.org/10.1002/cne.23786

Duncan, K., Tompary, A., & Davachi, L. (2014). Associative encoding and retrieval are predicted by functional connectivity in distinct hippocampal area CA1 pathways. *The Journal of Neuroscience: The Official Journal of the Society for Neuroscience*, *34*(34), 11188–11198. https://doi.org/10.1523/JNEUROSCI.0521-14.2014

Duvernoy, H. M. (2005). *The Human Hippocampus: Functional Anatomy, Vascularization and Serial Sections with MRI*. Springer Science & Business Media.

Gallyas, F. (1979). Silver Staining of Myelin by Means of Physical Development. *Neurological Research*, *1*(2), Article 2. https://doi.org/10.1080/01616412.1979.11739553

Gittins, R., & Harrison, P. J. (2004). Neuronal density, size and shape in the human anterior cingulate cortex: A comparison of Nissl and NeuN staining. *Brain Research Bulletin*, *63*(2), 155–160. https://doi.org/10.1016/j.brainresbull.2004.02.005

Gómez-Isla, T., Hollister, R., West, H., Mui, S., Growdon, J. H., Petersen, R. C., Parisi, J. E., & Hyman, B. T. (1997). Neuronal loss correlates with but exceeds neurofibrillary tangles in Alzheimer’s disease. *Annals of Neurology*, *41*(1), 17–24. https://doi.org/10.1002/ana.410410106

Gómez-Isla, T., Price, J. L., McKeel, D. W., Morris, J. C., Growdon, J. H., & Hyman, B. T. (1996). Profound loss of layer II entorhinal cortex neurons occurs in very mild Alzheimer’s disease. *The Journal of Neuroscience: The Official Journal of the Society for Neuroscience*, *16*(14), 4491–4500. https://doi.org/10.1523/JNEUROSCI.16-14-04491.1996

Hitti, F. L., & Siegelbaum, S. A. (2014). The hippocampal CA2 region is essential for social memory. *Nature*, *508*(7494), Article 7494. https://doi.org/10.1038/nature13028

Insausti, R., & Amaral, D. (2004). Hippocampal Formation. In *The Human Nervous System, Second Addition* (Second, pp. 871–914).

Insausti, R., Muñoz-López, M., & Insausti, A. M. (2023). The CA2 hippocampal subfield in humans: A review. *Hippocampus*, *33*(6), 712–729. https://doi.org/10.1002/hipo.23547

Insausti, R., Muñoz-López, M., Insausti, A. M., & Artacho-Pérula, E. (2017). The Human Periallocortex: Layer Pattern in Presubiculum, Parasubiculum and Entorhinal Cortex. A Review. *Frontiers in Neuroanatomy*, *11*, 84. https://doi.org/10.3389/fnana.2017.00084

Insausti, R., Tuñón, T., Sobreviela, T., Insausti, A. M., & Gonzalo, L. M. (1995). The human entorhinal cortex: A cytoarchitectonic analysis. *Journal of Comparative Neurology*, *355*(2), Article 2. https://doi.org/10.1002/cne.903550203

Kerchner, G. A., Deutsch, G. K., Zeineh, M., Dougherty, R. F., Saranathan, M., & Rutt, B. K. (2012). Hippocampal CA1 apical neuropil atrophy and memory performance in Alzheimer’s disease. *NeuroImage*, *63*(1), 194–202. https://doi.org/10.1016/j.neuroimage.2012.06.048

Kirwan, C. B., Jones, C. K., Miller, M. I., & Stark, C. E. L. (2007). High-resolution fMRI investigation of the medial temporal lobe. *Human Brain Mapping*, *28*(10), 959–966. https://doi.org/10.1002/hbm.20331

La Joie, R., Fouquet, M., Mézenge, F., Landeau, B., Villain, N., Mevel, K., Pélerin, A., Eustache, F., Desgranges, B., & Chételat, G. (2010). Differential effect of age on hippocampal subfields assessed using a new high-resolution 3T MR sequence. *NeuroImage*, *53*(2), 506–514. https://doi.org/10.1016/j.neuroimage.2010.06.024

Lehr, A. B., Kumar, A., Tetzlaff, C., Hafting, T., Fyhn, M., & Stöber, T. M. (2021). CA2 beyond social memory: Evidence for a fundamental role in hippocampal information processing. *Neuroscience and Biobehavioral Reviews*, *126*, 398–412. https://doi.org/10.1016/j.neubiorev.2021.03.020

Malykhin, N. V., Carter, R., Seres, P., & Coupland, N. J. (2010). Structural changes in the hippocampus in major depressive disorder: Contributions of disease and treatment. *Journal of Psychiatry & Neuroscience : JPN*, *35*(5), Article 5. https://doi.org/10.1503/jpn.100002

Mueller, S. G., Stables, L., Du, A. T., Schuff, N., Truran, D., Cashdollar, N., & Weiner, M. W. (2007). Measurement of hippocampal subfields and age-related changes with high resolution MRI at 4 T. *Neurobiology of Aging*, *28*(5), Article 5. https://doi.org/10.1016/j.neurobiolaging.2006.03.007

Niazi, M. K. K., Parwani, A. V., & Gurcan, M. N. (2019). Digital pathology and artificial intelligence. *The Lancet. Oncology*, *20*(5), e253–e261. https://doi.org/10.1016/S1470-2045(19)30154-8

Oliva, A. (2022). CA2 physiology underlying social memory. *Current Opinion in Neurobiology*, *77*, 102642. https://doi.org/10.1016/j.conb.2022.102642

Oliva, A., Fernandez-Ruiz, A., & Karaba, L. A. (2023). CA2 orchestrates hippocampal network dynamics. *Hippocampus*, *33*(3), 241–251. https://doi.org/10.1002/hipo.23495

Olsen, R. K., Carr, V. A., Daugherty, A. M., La Joie, R., Amaral, R. S. C., Amunts, K., Augustinack, J. C., Bakker, A., Bender, A. R., Berron, D., Boccardi, M., Bocchetta, M., Burggren, A. C., Chakravarty, M. M., Chételat, G., de Flores, R., DeKraker, J., Ding, S.-L., Geerlings, M. I., … Hippocampal Subfields Group. (2019). Progress update from the hippocampal subfields group. *Alzheimer’s & Dementia (Amsterdam, Netherlands)*, *11*, 439–449. https://doi.org/10.1016/j.dadm.2019.04.001

Oltmer, J., Rosenblum, E. W., Williams, E. M., Roy, J., Llamas-Rodriguez, J., Perosa, V., Champion, S. N., Frosch, M. P., & Augustinack, J. C. (2023). Stereology neuron counts correlate with deep learning estimates in the human hippocampal subregions. *Scientific Reports*, *13*(1), 5884. https://doi.org/10.1038/s41598-023-32903-y

Orban, G. A., & Vanduffel, W. (2007). Comment on Devlin and Poldrack. *NeuroImage*, *37*(4), 1057–1058; discussion 1066-1068. https://doi.org/10.1016/j.neuroimage.2007.02.009

Palomero-Gallagher, N., Kedo, O., Mohlberg, H., Zilles, K., & Amunts, K. (2020). Multimodal mapping and analysis of the cyto- and receptorarchitecture of the human hippocampus. *Brain Structure & Function*, *225*(3), 881–907. https://doi.org/10.1007/s00429-019-02022-4

Passingham, R. E. (2007). Commentary on Devlin and Poldrack. *NeuroImage*, *37*(4), 1055–1056; discussion 1066-1068. https://doi.org/10.1016/j.neuroimage.2007.02.010

Perosa, V., Oltmer, J., Munting, L. P., Freeze, W. M., Auger, C. A., Scherlek, A. A., van der Kouwe, A. J., Iglesias, J. E., Atzeni, A., Bacskai, B. J., Viswanathan, A., Frosch, M. P., Greenberg, S. M., & van Veluw, S. J. (2022). Perivascular space dilation is associated with vascular amyloid-β accumulation in the overlying cortex. *Acta Neuropathologica*, *143*(3), 331–348. https://doi.org/10.1007/s00401-021-02393-1

Rosene, D. L., & Van Hoesen, G. W. (1987). The Hippocampal Formation of the Primate Brain. In E. G. Jones & A. Peters (Eds.), *Cerebral Cortex: Further Aspects of Cortical Function, Including Hippocampus* (pp. 345–456). Springer US. https://doi.org/10.1007/978-1-4615-6616-8_9

Saunders, R. C., Rosene, D. L., & Van Hoesen, G. W. (1988). Comparison of the efferents of the amygdala and the hippocampal formation in the rhesus monkey: II. Reciprocal and non-reciprocal connections. *The Journal of Comparative Neurology*, *271*(2), 185–207. https://doi.org/10.1002/cne.902710203

Steve, T. A., Yasuda, C. L., Coras, R., Lail, M., Blumcke, I., Livy, D. J., Malykhin, N., & Gross, D. W. (2017). Development of a histologically validated segmentation protocol for the hippocampal body. *NeuroImage*, *157*, 219–232. https://doi.org/10.1016/j.neuroimage.2017.06.008

Swanson, L. W. (2003). *Brain Architecture: Understanding the Basic Plan*. Oxford University Press.

Taube, J. S., Muller, R. U., & Ranck, J. B. (1990). Head-direction cells recorded from the postsubiculum in freely moving rats. I. Description and quantitative analysis. *The Journal of Neuroscience: The Official Journal of the Society for Neuroscience*, *10*(2), 420–435. https://doi.org/10.1523/JNEUROSCI.10-02-00420.1990

Toga, A. W., & Thompson, P. M. (2007). What is where and why it is important. *NeuroImage*, *37*(4), 1045–1049; discussion 1066-1068. https://doi.org/10.1016/j.neuroimage.2007.02.018

Tukker, J. J., Tang, Q., Burgalossi, A., & Brecht, M. (2015). Head-Directional Tuning and Theta Modulation of Anatomically Identified Neurons in the Presubiculum. *The Journal of Neuroscience: The Official Journal of the Society for Neuroscience*, *35*(46), 15391–15395. https://doi.org/10.1523/JNEUROSCI.0685-15.2015

Turner, R., & Geyer, S. (2014). Comparing like with like: The power of knowing where you are. *Brain Connectivity*, *4*(7), 547–557. https://doi.org/10.1089/brain.2014.0261

Tzourio-Mazoyer, N., Hervé, P. Y., & Mazoyer, B. (2007). Neuroanatomy: Tool for functional localization, key to brain organization. *NeuroImage*, *37*(4), 1059–1060; discussion 1066-1068. https://doi.org/10.1016/j.neuroimage.2007.02.007

Van Essen, D. C., & Dierker, D. (2007). On navigating the human cerebral cortex: Response to “in praise of tedious anatomy.” *NeuroImage*, *37*(4), 1050–1054; discussion 1066-1068. https://doi.org/10.1016/j.neuroimage.2007.02.021

Vogt, C., & Vogt, O. (1919). *Allgemeine Ergebnisse unserer Hirnforschung*. J.A. Barth.

Waisman, A., Norris, A. M., Elías Costa, M., & Kopinke, D. (2021). Automatic and unbiased segmentation and quantification of myofibers in skeletal muscle. *Scientific Reports*, *11*(1), 11793. https://doi.org/10.1038/s41598-021-91191-6

Wang, J., & Barbas, H. (2018). Specificity of Primate Amygdalar Pathways to Hippocampus. *Journal of Neuroscience*, *38*(47), Article 47. https://doi.org/10.1523/JNEUROSCI.1267-18.2018

Wang, L., Swank, J. S., Glick, I. E., Gado, M. H., Miller, M. I., Morris, J. C., & Csernansky, J. G. (2003). Changes in hippocampal volume and shape across time distinguish dementia of the Alzheimer type from healthy aging. *NeuroImage*, *20*(2), 667–682. https://doi.org/10.1016/S1053-8119(03)00361-6

West, M. J., Kawas, C. H., Martin, L. J., & Troncoso, J. C. (2000). The CA1 region of the human hippocampus is a hot spot in Alzheimer’s disease. *Annals of the New York Academy of Sciences*, *908*, 255–259. https://doi.org/10.1111/j.1749-6632.2000.tb06652.x

Williams, E. M., Rosenblum, E. W., Pihlstrom, N., Llamas-Rodríguez, J., Champion, S., Frosch, M. P., & Augustinack, J. C. (2023). Pentad: A reproducible cytoarchitectonic protocol and its application to parcellation of the human hippocampus. *Frontiers in Neuroanatomy*, *17*. https://www.frontiersin.org/articles/10.3389/fnana.2023.1114757

Winterburn, J. L., Pruessner, J. C., Chavez, S., Schira, M. M., Lobaugh, N. J., Voineskos, A. N., & Chakravarty, M. M. (2013). A novel in vivo atlas of human hippocampal subfields using high-resolution 3 T magnetic resonance imaging. *NeuroImage*, *74*, 254–265. https://doi.org/10.1016/j.neuroimage.2013.02.003

Wisse, L. E. M., Daugherty, A. M., Olsen, R. K., Berron, D., Carr, V. A., Stark, C. E. L., Amaral, R. S. C., Amunts, K., Augustinack, J. C., Bender, A. R., Bernstein, J. D., Boccardi, M., Bocchetta, M., Burggren, A., Chakravarty, M. M., Chupin, M., Ekstrom, A., de Flores, R., Insausti, R., … la Joie, R. (2017). A harmonized segmentation protocol for hippocampal and parahippocampal subregions: Why do we need one and what are the key goals? *Hippocampus*, *27*(1), Article 1. https://doi.org/10.1002/hipo.22671

Wisse, L. E. M., Gerritsen, L., Zwanenburg, J. J. M., Kuijf, H. J., Luijten, P. R., Biessels, G. J., & Geerlings, M. I. (2012). Subfields of the hippocampal formation at 7T MRI: In vivo volumetric assessment. *NeuroImage*, *61*(4), Article 4. https://doi.org/10.1016/j.neuroimage.2012.03.023

Yushkevich, P. A., Amaral, R. S. C., Augustinack, J. C., Bender, A. R., Bernstein, J. D., Boccardi, M., Bocchetta, M., Burggren, A. C., Carr, V. A., Chakravarty, M. M., Chételat, G., Daugherty, A. M., Davachi, L., Ding, S.-L., Ekstrom, A., Geerlings, M. I., Hassan, A., Huang, Y., Iglesias, J. E., … Hippocampal Subfields Group (HSG). (2015). Quantitative comparison of 21 protocols for labeling hippocampal subfields and parahippocampal subregions in in vivo MRI: Towards a harmonized segmentation protocol. *NeuroImage*, *111*, 526–541. https://doi.org/10.1016/j.neuroimage.2015.01.004

Zeineh, M. M., Holdsworth, S., Skare, S., Atlas, S. W., & Bammer, R. (2012). Ultra-high resolution diffusion tensor imaging of the microscopic pathways of the medial temporal lobe. *NeuroImage*, *62*(3), 2065–2082. https://doi.org/10.1016/j.neuroimage.2012.05.065

**Figure 1.** **Microscopic View of Neurons in Subiculum, Prosubiculum and CA1.** Panels A’-C’ depict neuron tracings of panels A, B and C, respectively. Nissl stained sections with microscopic magnification (case 4 (A-C), case 5 (D-F), case 6 (G-I), case 7 (J-L), case 8 (M-O)) depict pyramidal neurons from the Sub, ProS and CA1 from the same histologic section. Note that the subicular pyramidal neurons exhibit a relatively large size (A’, A, D, G, J, and M). ProS shows an intermingling of small and large pyramidal neurons; the small neurons show a particularly narrow morphology (B’, B, E, H, K, and N). CA1 pyramidal neurons display a large triangular but ovoid shape (C’, C, F, I, L, O). Each panel shows the same magnification and field of view to discern neuron size dissimilarities. The ProS neurons appear less chromophilic (B, E, H) than their Sub and CA1 counterparts. Magnification bar = 50 µm.

**Figure 2. Quantitative measures for neuron size in Sub, ProS and CA1.**

ProS neurons were significantly different (smaller) than Sub and CA1 neurons in size (n=400 per subfield, Kruskall-Wallace, p < .0001, Dunn’s multiple comparisons, p < .0001, p < .0001). ProS neurons averaged 14.31 μm at the base of the pyramid, while CA1 neurons averaged 15.63 μm and Sub neurons averaged 15.57 μm. ProS neurons were the smallest but CA1 showed the largest variation. Whisker bars show the range and horizontal lines within the box represent the median.

**Figure 3. Mesoscopic views of ProS.** The images show ProS in all ten samples at the mesoscopic view (Same order as **Table 1**). This view is optimal to distinguish differences between ProS and its surrounding subfields, showing layers, and to some degree, neurons. Panels C, D, E, H, and J show lightly stained cells. Panels B, E, H and I demonstrate clustered cells (asterisks) and E, H and I highlight cell sparse zones (arrows). Magnification bar = 1 mm.

**Figure 4.** **Macroscopic views of Prosubiculum.** Panels A – J show Nissl stained histologic sections at a birds eye view and parcellated for ProS (same order as Fig. 3 and Table 1). Note that ProS consistently resides between CA1 and Sub but varies in size and relative location (black boundary lines). The relative location shifts slightly along the allocortical ribbon depending on the particular case; for example, D and E sit more medially than other cases. CA1 routinely displays jagged inner border for the pyramidal stratum (arrowheads in B). Magnification bar = 2mm.

**Figure 5. Gross Dissection of a Human Hippocampus Specimen**

A and B depict images of a human hippocampus from a superior view (with the anterior end of hippocampus at top). Gross morphological landmarks are annotated, pes (asterisks), gyrus intralimbicus (GI) in A. B) shows coronal planes of cut (horizontal lines) that annotate the approximate the locations of the following: a) *genu*, b) *genu-pes*, c) *pes*, d) *pes-DG*, e) *full DG*, f) *separated DG*, g) *x-region*, i) *anterior body*, j) *mid-body*, k) *posterior body*. The territory between lines a and h encompasses the hippocampal head and between h-k resides the hippocampal body. C) shows respective coronal schemata (i.e., genu, pes, full DG and anterior body). In drawings of C, black lines represent the tissue edge, gray lines represent the pyramidal layer, dark blue lines denote the dentate gyrus while the dotted lines illustrate the gray-white-matter border. Abbreviations: Ant. = Anterior; Post. = Posterior; GI = gyrus intralimbicus

**Figure 6. Matrices show representations of four cytoarchitectural features along the hippocampal axis.** Matrices collectively depict ProS traits: small neurons, lightly stained neurons, clustered neurons and a cell free zone in 10 postmortem samples. Architectonic feature data compiled from 180 histologic sections (18 histologic sections per case) throughout the anterior to posterior extent of the hippocampus. Matrices (panels A-J) follow the same order arrangement as **Table 1**, **Figures 3, 4**. Color code for architectonic features indicates small neurons (blue), lightly stained cells (pink), clustered cells (orange) and a cell free zone (green). ProS began anteriorly at the level of genu or genu pes. Small cells were the most common trait, observed in every case. This frequency in our sample set was present 93% of Nissl sections for ProS, evident by the dominant blue color in the ten matrices. The remaining frequencies followed with lightly stained cells at 34%, clustered cells at 35%, and sparse zone at only 19% of Nissl sections with ProS. The matrices demonstrate the heterogeneity of features and an example for the variability of the human brain.

**Figure 7. Tau immunostaining in ProS.**

A) shows a photomacrograph for p-tau immunostaining from case 9 (tau severity is Braak stage II). Phosphorylated-tau antibody is CP13 (AT8 equivalent) and phosphorylated at serine 202. Panels B-D shows neurons affected with neurofibrillary tangles from Sub, ProS and CA1, respectively. Panel C aptly shows the differential in neuron (tangle) size for ProS, with one large tangle but several other small tangles. Though p-tau is pathologic, it still shows the relative size and highlights the intermingling for neuron size. Magnification bar in A = 2.5 mm; in D= 100 μm.
