# Supplementary material for: The prosubiculum in the human hippocampus: A rostrocaudal, feature-driven, and systematic approach
Source: J Comp Neurol. Author manuscript; Available in PMC 2025 Mar 1. (PMC11060218; doi:10.1002/cne.25604)
Supplement: Supinfo [file NIHMS1973107-supplement-Supinfo.zip › jcn-23-0166-manifest.html]

Manifest file for export jcn-23-0166-20240229022704


|  |  |
| --- | --- |
| File name: jcn-23-0166-20240229022704 | |
| Export Date: 29-Feb-2024 | |
| Output Format: XML (ScholarOne DTD) | |
| jcn-23-0166-20240229022704/doc/Rosenblum\_Manuscript\_20240112\_clean.docx | Version 1.1 |
| jcn-23-0166-20240229022704/graphic/Figure\_1.png | Version 1.0 |
| jcn-23-0166-20240229022704/graphic/Figure\_2.pdf | Version 1.0 |
| jcn-23-0166-20240229022704/graphic/Figure\_3.png | Version 1.0 |
| jcn-23-0166-20240229022704/graphic/Figure\_4.png | Version 1.0 |
| jcn-23-0166-20240229022704/graphic/Figure\_5.png | Version 1.0 |
| jcn-23-0166-20240229022704/graphic/Figure\_6.png | Version 1.0 |
| jcn-23-0166-20240229022704/graphic/Figure\_7.png | Version 1.0 |
| jcn-23-0166-20240229022704/graphic/Rosenblum\_table\_1.docx | Version 1.0 |
| jcn-23-0166-20240229022704/graphic/Rosenblum\_Table\_2.docx | Version 1.0 |
| jcn-23-0166-20240229022704/doc/Graphical\_abstract.png | Version 1.0 |
| jcn-23-0166-20240229022704/doc/Graphical\_Abstract.docx | Version 1.0 |
| jcn-23-0166-20240229022704/pdf/jcn-23-0166.pdf |  |
| jcn-23-0166-20240229022704/jcn-23-0166-metadata.xml |  |
| jcn-23-0166-20240229022704/s1.dtd |  |
| manifest.html | This document |
