# Supplementary material for: The prosubiculum in the human hippocampus: A rostrocaudal, feature-driven, and systematic approach
Source: J Comp Neurol. Author manuscript; Available in PMC 2025 Mar 1. (PMC11060218; doi:10.1002/cne.25604)
Supplement: Supinfo [file NIHMS1973107-supplement-Supinfo.zip › Rosenblum_Table_2.docx]

| **Author, Year** | **Method** | **Includes ProS?** | **How is ProS classified** |
| --- | --- | --- | --- |
| Vogt and Vogt, 1919 | Nissl  Myelin | Yes | Not described |
| Lorente De No, 1934 | Golgi  Nissl | Yes | **Golgi:** lack of the stratum radiatum or  lacunosum, and silver staining observation |
| Rosene and Van Hoesen, 1987 | Nissl  Timms  Acetylcholinesterase | Yes | **Nissl:** Small neurons, clustering of superficial neurons, ending of stratum radiatum  **Ache:** high Ache staining in ProS |
| Insausti and Amaral, 2004 | Nissl  Timms | No | Not included,  Not described |
| Duvernoy, 2005 | India Ink  Bodian’s Silver | Yes | Not described |
| Ding, 2013 | Acetylcholinesterase  Neurotensin  Tyrosine Hydroxylase  # | Yes | **IHC:** Higher expression of acetylcholinesterase, neurotensin,  and tyrosine hydroxylase |
| Adler et al., 2014 | Luxol fast blue + Cresyl violet | No | Not included,  Not described |
| Ding and Van Hoesen, 2015 | Nissl  NeuN (ab)  Parvalbumin (ab) Calbindin (ab) | Yes | **NeuN:** Small neurons, superior clumping |
| Steve et al. 2017 | Cresyl violet + Luxol fast blue | No | Not included,  Not described |
| Palomero-Gallagher et al. 2019 | Receptor autoradiography | Yes | Lack of the stratum radiatum, appearance of deep, large subiculum like pyramidal neurons,  Superficial cell clumping, differences in receptors |
| Williams et al., 2023 | Nissl | Yes | **Nissl:** Small, lightly stained cells with superior clumping |

**Table 2. Summary of ProS parcellations from previous studies.**

Some studies did not include ProS in their parcellations and have been denoted as “Not Included.” Other studies include ProS in their parcellations but did not describe the traits (or criteria) used to parcellate ProS, noted as “ProS traits not described.” The symbol + denotes double staining. # denotes the study used other stains. See bibliography for further details about references. Abbreviations: ab = antibody.
