# Supplementary material for: The prosubiculum in the human hippocampus: A rostrocaudal, feature-driven, and systematic approach
Source: J Comp Neurol. Author manuscript; Available in PMC 2025 Mar 1. (PMC11060218; doi:10.1002/cne.25604)
Supplement: Supinfo [file NIHMS1973107-supplement-Supinfo.zip › Graphical_Abstract.docx]

Our findings provide evidence that the prosubiculum is an independent hippocampal subfield based on its unique cytoarchitecture. Small neurons constitute the defining feature of prosubiculum, while the other traits, lightly stained neurons, clustered neurons and a cell sparse zone, appear heterogeneously.
