# Supplementary figures and images for: The prosubiculum in the human hippocampus: A rostrocaudal, feature-driven, and systematic approach
Source: J Comp Neurol. Author manuscript; Available in PMC 2025 Mar 1. (PMC11060218; doi:10.1002/cne.25604)

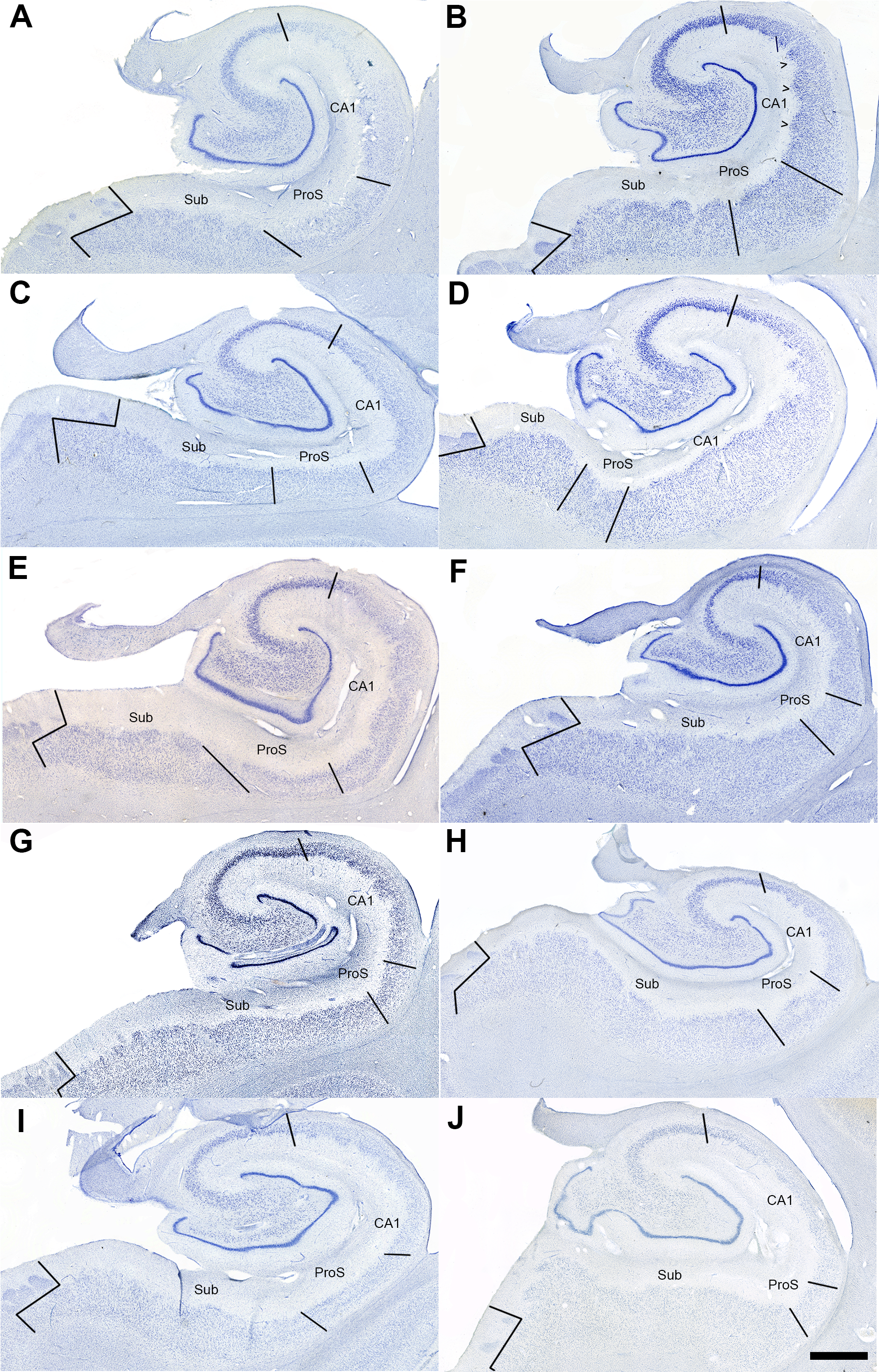

Supplement: Supinfo [file NIHMS1973107-supplement-Supinfo.zip › Figure_4.png]

Neuron width at base,  $\mu\text{m}$

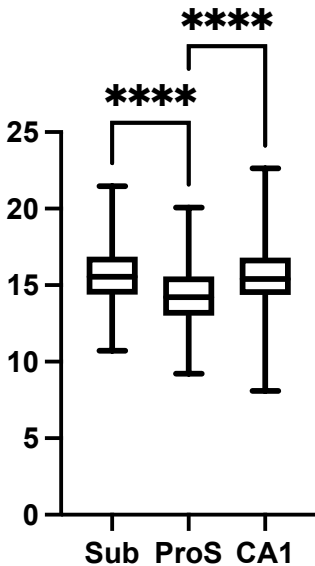

Supplement: Supinfo [file NIHMS1973107-supplement-Supinfo.zip › Figure_2.pdf]

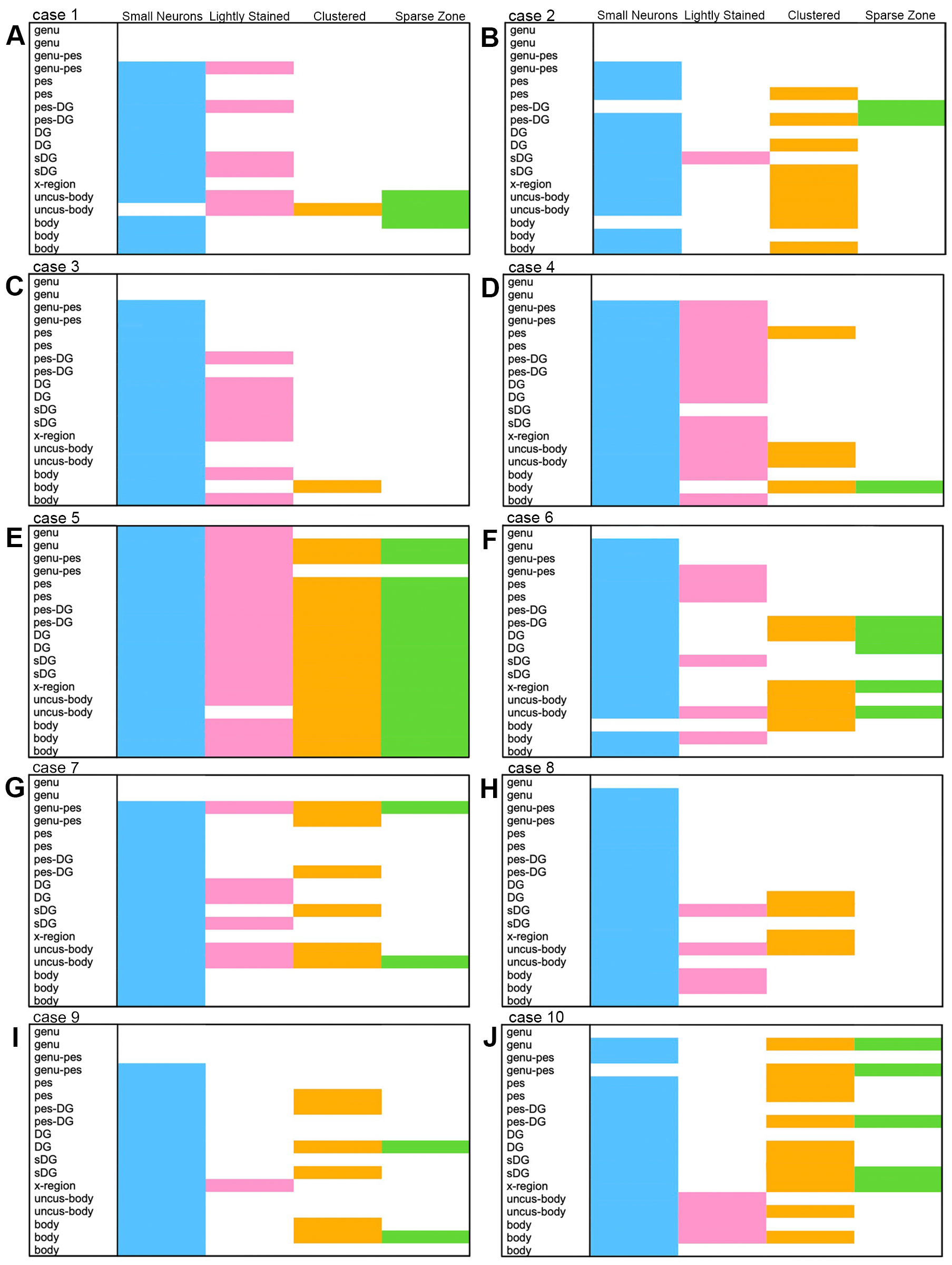

Supplement: Supinfo [file NIHMS1973107-supplement-Supinfo.zip › Figure_6.png]

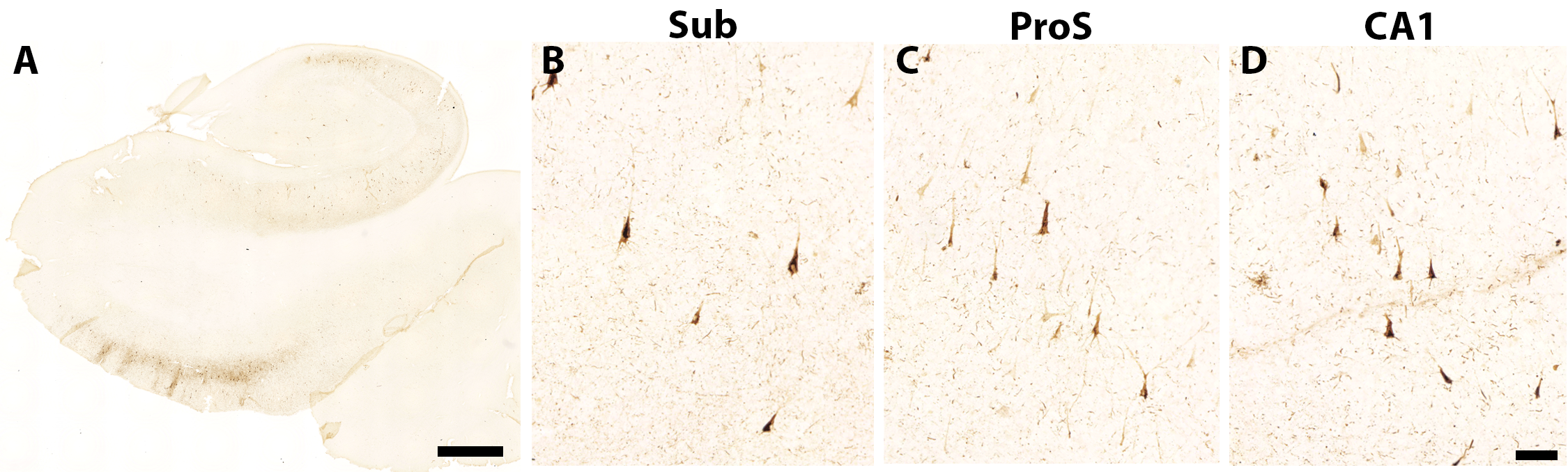

Supplement: Supinfo [file NIHMS1973107-supplement-Supinfo.zip › Figure_7.png]

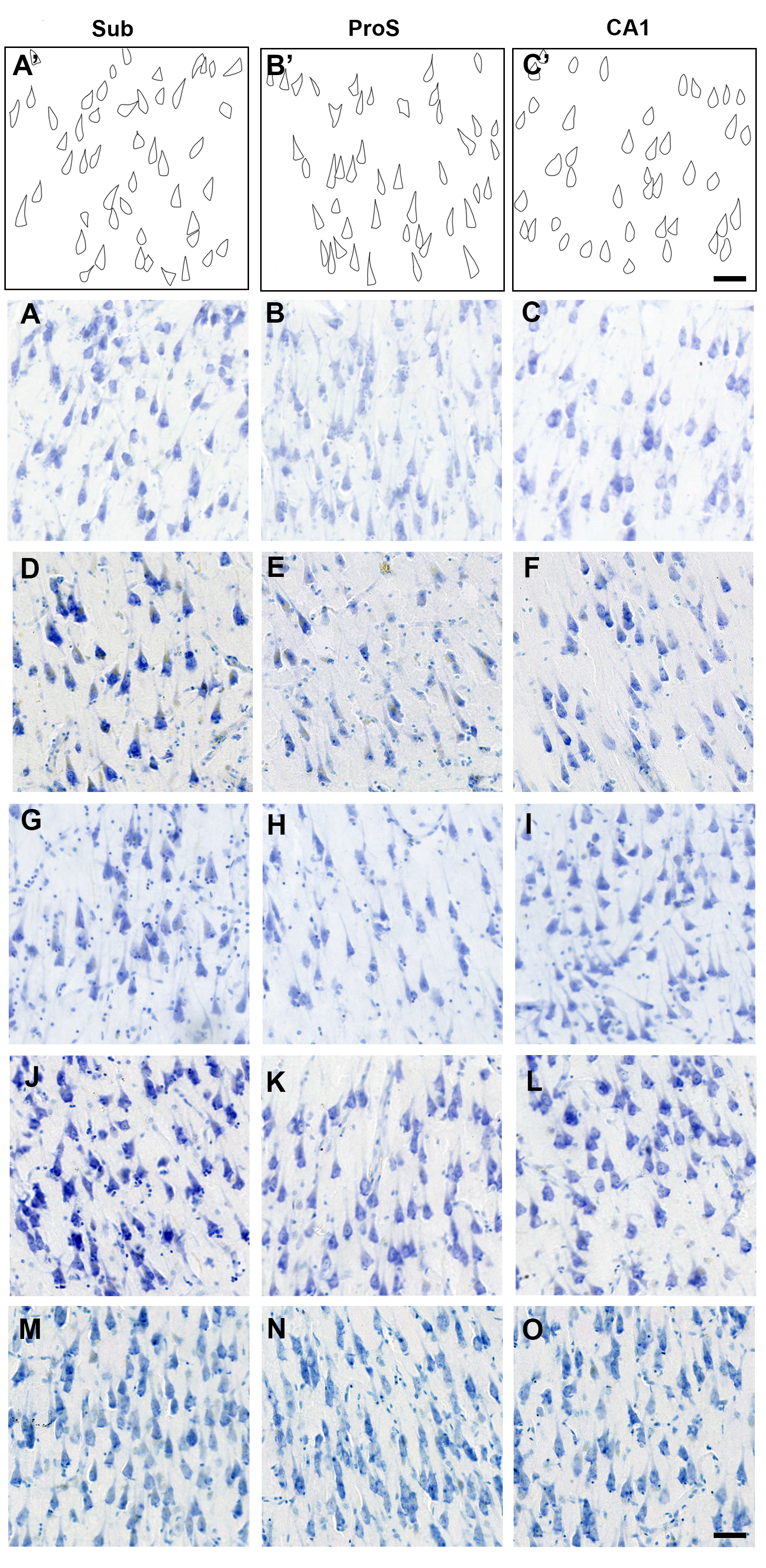

Supplement: Supinfo [file NIHMS1973107-supplement-Supinfo.zip › Figure_1.png]

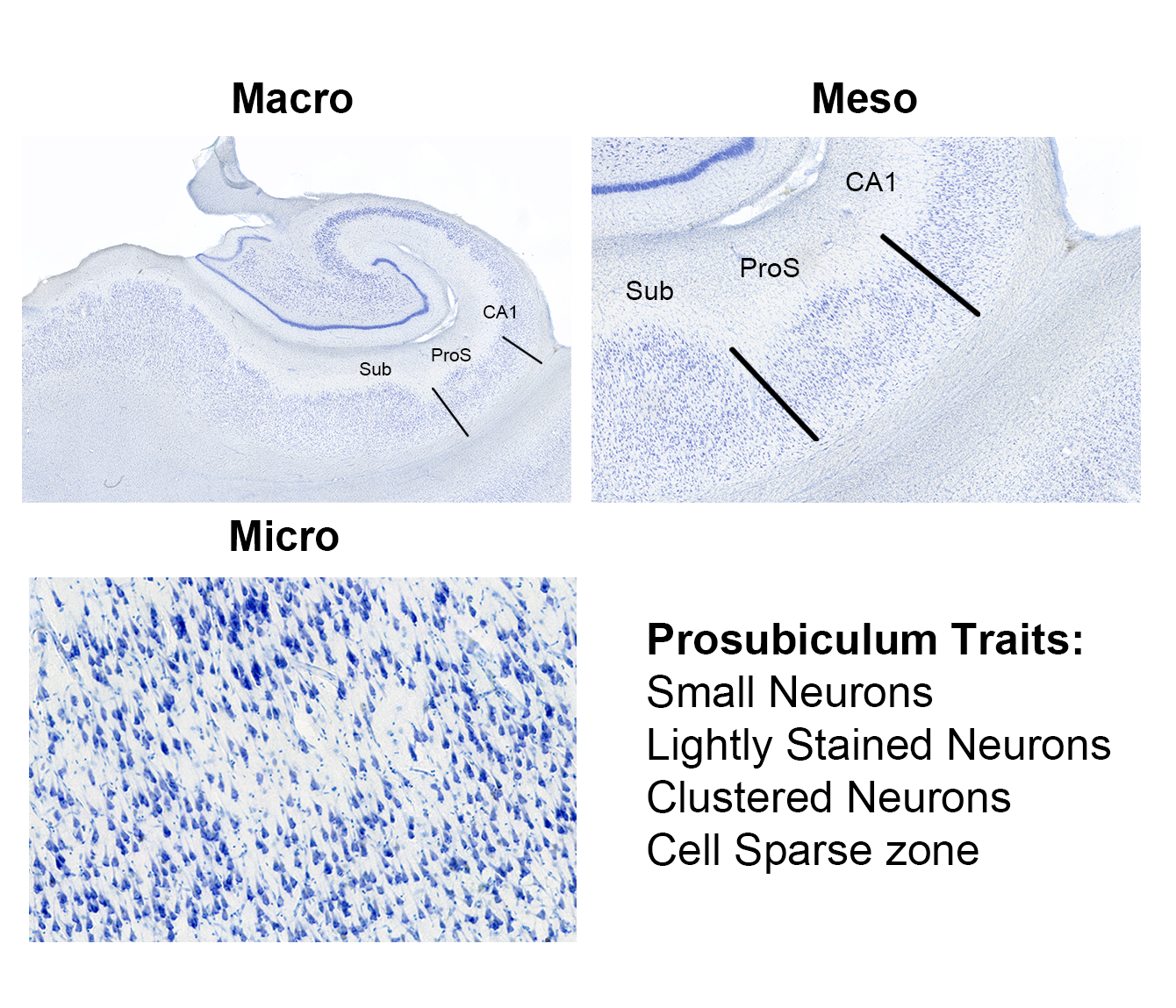

Supplement: Supinfo [file NIHMS1973107-supplement-Supinfo.zip › Graphical_abstract.png]

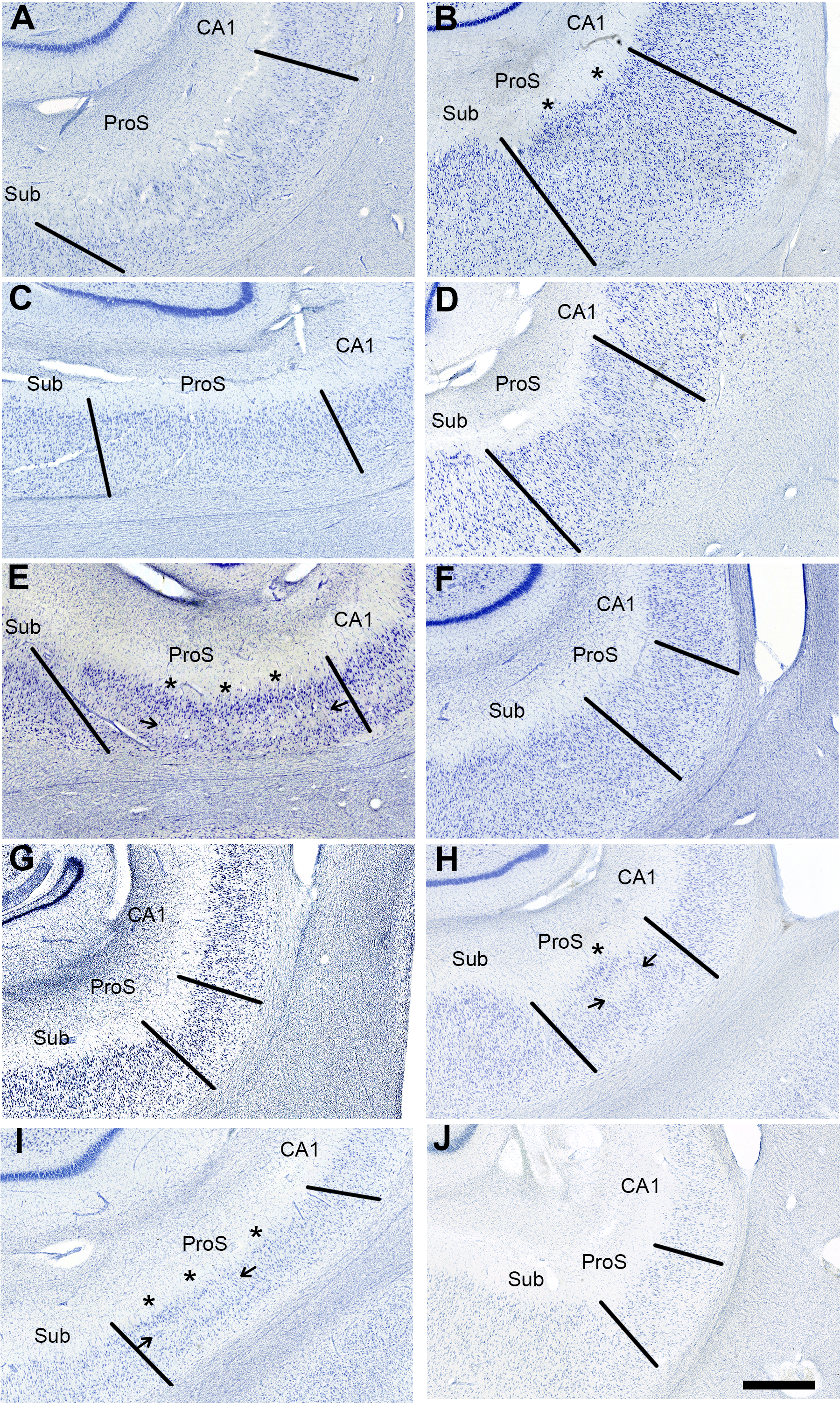

Supplement: Supinfo [file NIHMS1973107-supplement-Supinfo.zip › Figure_3.png]

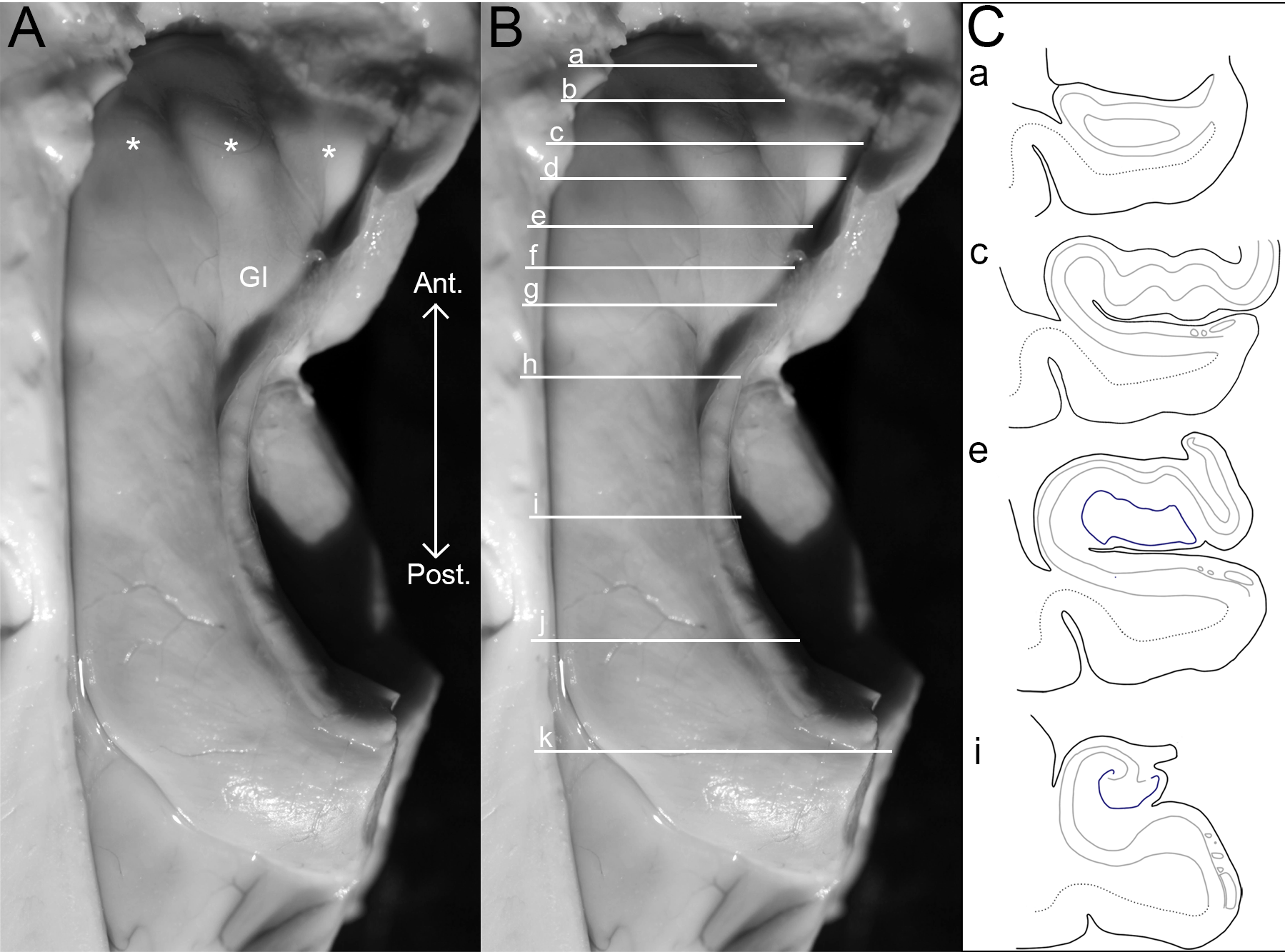

Supplement: Supinfo [file NIHMS1973107-supplement-Supinfo.zip › Figure_5.png]
